# MRSA: the transmission paradigm investigated

**DOI:** 10.1186/1753-6561-5-S6-O86

**Published:** 2011-06-29

**Authors:** G Moore, B Cookson, R Jackson, A Kearns, J Singleton, D Smyth, APR Wilson

**Affiliations:** 1 University College London Hospitals, London, UK; 2 Health Protection Agency, London, UK; 3 Royal Free Hampstead NHS Trust, London, UK

## Introduction / objectives

Variability in MRSA policies and procedures reflects a need for more information regarding the relative contribution and clinical importance of the different modes of MRSA transmission.

## Methods

A prospective 1 year study was conducted within the ICU of two UK hospitals. Conventional sampling techniques were used to recover MRSA from the air and from high contact sites located within the ward environment. All patients were screened for MRSA on admission and weekly during their stays. Samples from other sites were cultured when clinically indicated. MRSA was considered nosocomially acquired if detected more than 48 h after admission. Possible transmission routes from donor to recipient via the hands of staff, the air or environmental surfaces were identified. Focused molecular typing via PFGE was used to explore these pathway hypotheses.

## Results

There were 2654 admissions. 175 patients were positive for MRSA on admission whilst 78 acquired MRSA during their stay. MRSA was isolated from 208 of 18,596 surfaces, from 56 of 859 air samples and from the hands of staff on 59 of 4191 sampling occasions. 28 acquisition events occurred in ICU A. Only those involving an unusual PFGE pulsotype (i.e. one not recognised as being common and widespread within the UK hospital environment) were selected for further study. The likely source of 9 colonizations was identified and of the tested hypotheses, 7 implicated poor hand hygiene, 4 inadequate cleaning and 1 airborne transmission. In ICU B a higher level of endogenous and/or endemic MRSA meant evidence for patient-patient cross-transmission was more limited.

## Conclusion

To help minimise the spread of MRSA within an ICU, hand hygiene must be coupled with effective routine environmental cleaning.

## Disclosure of interest

None declared.

